# The translationally controlled tumor protein TCTP is involved in cell cycle progression and heat stress response in the bloodstream form of *Trypanosoma brucei*

**DOI:** 10.15698/mic2018.10.652

**Published:** 2018-08-24

**Authors:** Borka Jojic, Simona Amodeo, Torsten Ochsenreiter

**Affiliations:** 1 Institute of Cell Biology, University of Bern, Bern, Switzerland.; 2 Graduate School for Cellular and Biomedical Sciences, University of Bern, Bern, Switzerland.

**Keywords:** TCTP, Trypanosoma brucei, mitochondria, heat stress response, acidocalcisomes

## Abstract

The translationally controlled tumor protein TCTP, is a universally conserved protein that seems to be of essential function in all systems tested so far. TCTP is involved in a multitude of cellular functions including cell cycle control, cell division, apoptosis and many more. The mechanism of how TCTP is involved in most of these functions remains elusive. Here we describe that TCTP is a cytoplasmic protein involved in cell cycle regulation and heat stress response in the bloodstream form of *Trypanosoma brucei*.

## INTRODUCTION

*Trypanosoma brucei* is a single celled protozoan parasite and the causative agent of human African sleeping sickness and Nagana in cattle. The parasite belongs to the Excavata, an eukaryotic supergroup only distantly related to the mainstream model systems within the Opisthokonta and as such represents a versatile model to study universally conserved proteins and their potential functions in biology.

The translationally controlled tumor protein (TCTP) is highly conserved among eukaryotes and is involved in a large variety of processes like cell growth and development, the cell cycle, apoptosis and the protection against cellular stresses, including oxidative stress and heat stress 1,2,3,4,5,6,7,8. Moreover, several interacting/binding partners such as the elongation factor eEF-1alpha 9, tubulin 10, calcium 11 or Na^+^/ K^+^-ATPase 12 have been identified.

We recently showed that *T. brucei* contains two isoforms TCTP1 and TCTP2 that are exclusively expressed in the procyclic form (PCF) and bloodstream form (BSF) parasites, respectively 13. TCTP1 and TCTP2 have identical 5’UTRs and ten nucleotide changes in the open reading frames (ORFs) that lead to five amino acid changes. The exclusive expression is based on the different nucleotide composition of the 3'UTRs of the two isoforms, responsible for the different mRNA stabilities 13. TCTP1 is localized in the cytoplasm of PCF cells and loss of the protein causes a growth defect and leads to several phenotypes including a decrease in number and enlargement of acidocalcisomes (ACs) as well as changes in mitochondrial morphology. Furthermore, the cells depleted of TCTP display asymmetrical cell divisions leading to the accumulation of shorter "tadpole" like cells 13. While the cytoplasmic localization of TCTP has been described in several systems, the protein is also associated with other structures in the cell. In HeLa cells for example TCTP was mostly found in the nucleus, where it seems to be involved in anti-apoptotic activities since depletion by RNAi led to massive cell death by apoptosis 14. Also, associations with mitochondria have previously been demonstrated, albeit mainly under stress conditions 15,16,17. TCTP has previously also been described to be secreted and involved in inflammatory response through the release of histamine. How TCTP is released out of the cells remains enigmatic, but interestingly a similar observation was recently reported in trypanosome infected tsetse flies were TCTP might be involved in manipulating the microbiota of the fly 18.

As a consequence of harboring only one mitochondrion per cell with a singular mitochondrial genome known as the kinetoplast, trypanosomes display a synchronized mitochondrial and nuclear genome replication and segregation 19. Mitochondrial genome replication initiates prior to nuclear DNA replication and also the segregation of the replicated mitochondrial genome occurs prior to mitosis. A wild type BSF population contains about 80 - 85% cells with one kinetoplast and one nucleus (1K1N), 10 - 15% cells with two kinetoplasts and one nucleus (2K1N) and up to 5% cells with two kinetoplasts and two nuclei (2K2N) stage. Here we provide the first evidence for the localization of TCTP in BSF cells and its requirement for proper cytokinesis and heat stress response in the mammalian infective form of the parasite.

## RESULTS

### TCTP2 localization

**Figure 1:**
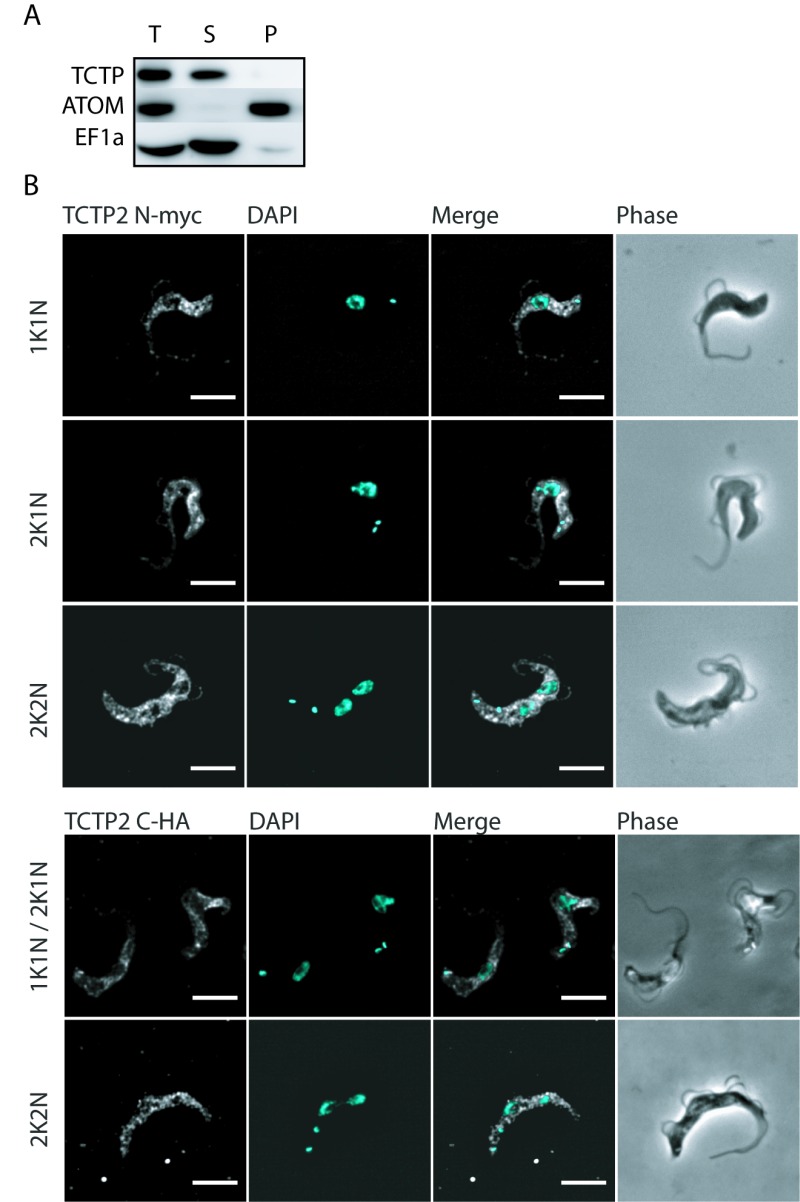
FIGURE 1: Localization of TCTP in bloodstream trypanosomes. **(A)** Western blot of BSF cells extracted with 0.025% digitonin. Total cellular extract (T), supernatant (S) and pellet (P) fractions were analysed by western blot decorated with antibodies against TCTP, ATOM and EF1α. **(B)** Immunofluorescence images of BSF cell expressing N-terminally myc-tagged TCTP2 or C-terminal HA-tagged TCTP2. Myc-TCTP2 was detected with anti-myc antibody (white). The DNA is detected by DAPI (cyan). K, kinetoplast DNA; N = nuclear DNA; Phase, phase contrast microscopy; scale bars: 5 µm.

In biochemical fractionations using digitonin and differential centrifugation followed by western blotting with the previously described anti-TCTP antibody, the protein is localized to the cytoplasmic fraction (Figure 1A). Since the antibody did not provide specificity in immunofluorescence microscopy, we tagged *TCTP2* N- (myc) and C-terminally (triple HA) to evaluate its localization using anti-myc and anti-HA antibodies (Figure 1B). These experiments support the biochemical analysis and show that the ectopically expressed N- or C-terminally tagged TCTP2 is predominantly localized in the cytoplasm with a distinct depletion of the TCTP2 signal in the region of the nucleus (Figure 1B). Furthermore, the protein does not seem to change its localization during the cell cycle (Figure 1B). DAPI was used to stain nuclear and kinetoplast DNA.

### TCTP is required for normal cell growth and cell cycle progression

**Figure 2:**
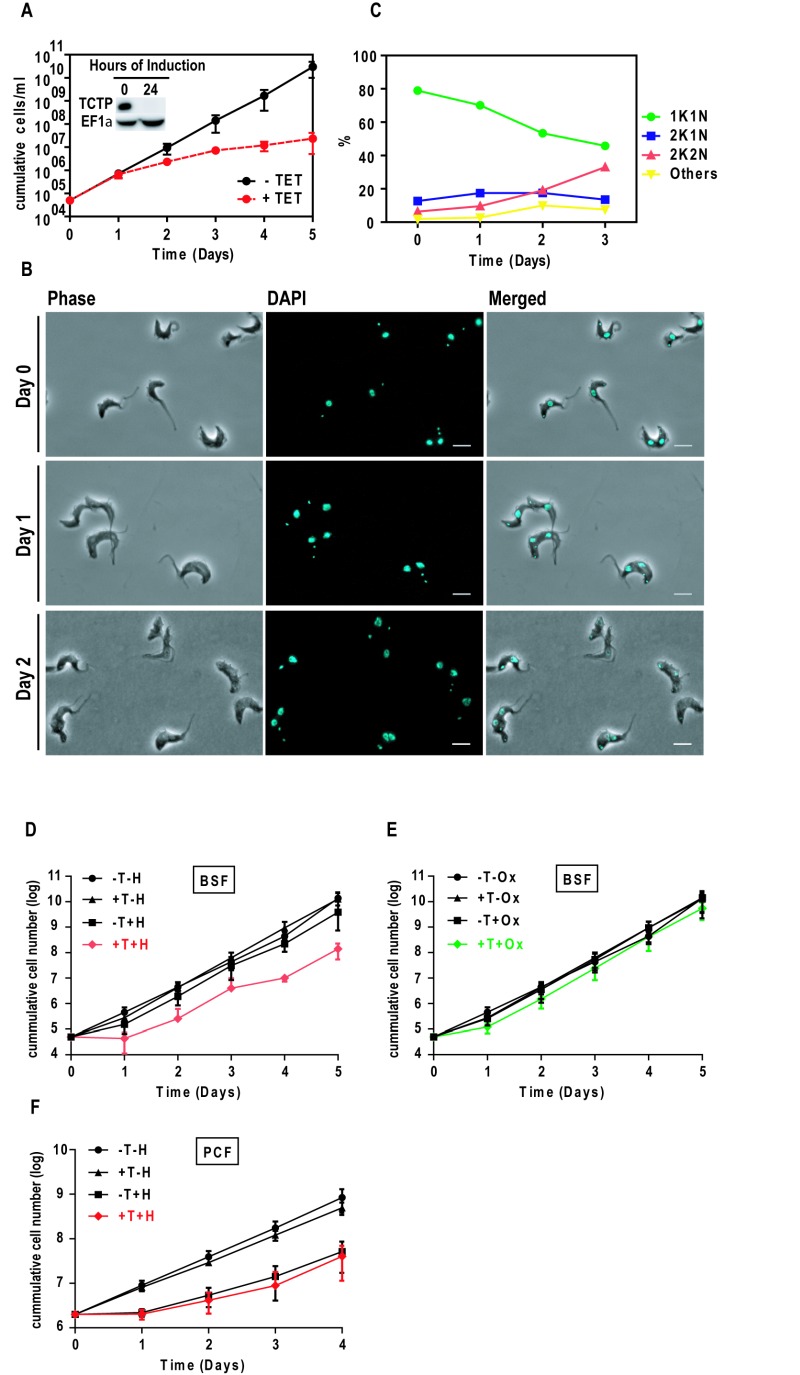
FIGURE 2: Effect of TCTP depletion, heat and oxidative stress in bloodstream trypanosomes. **(A)** Growth of cells with or without tet was monitored for five consecutive days. Bars represent standard deviation of four independent experiments. Inset: western blot showing TCTP downregulation upon 24 hours of RNAi induction. **(B)** Phase contrast and DAPI images of non-induced (Day 0) and induced (Day 1 and Day 2) bloodstream trypanosomes showing the effect of TCTP RNAi in cell morphology and cell cycle progression. **(C) **K-N counts in percentage following TCTP RNAi (n ≥ 100). Scale bars: 5 µm. **(D, E, F)** Monitoring growth of *T. brucei* upon *TCTP* RNAi followed by heat/oxidative stress. **(D)** BSF trypanosomes were heat shocked (+tet+heat shock, +T+H, red) at 42° C for one hour or **(E)** incubated with 50 µM sodium hydrogen arsenate (+T+oxidative stress, +T+Ox, green) for three hours, or **(F)** PCF cells were subjected to 45 minutes heat shock at 41°C. Subsequently the cells were washed in PBS and cultured again in HMI-9+10%FCS. Cell recovery was monitored for five consecutive days. Values represent averages from three independent experiments and error bars indicate standard deviation. Non-induced cells (-tet, -T), 24 hours induced (+tet, +T) cells and non-induced cells but stressed respectively with sodium hydrogen arsenate (-T+Ox) or heat shock (-T+H) were used as controls.

An inducible *TCTP* RNAi construct targeting the *TCTP *open reading frames was stably integrated in a BSF *T. brucei* cell line. We targeted the *TCTP* ORF in order to compare the results to the previously published phenotype in the PCF, where we also targeted the *TCTP* ORF. Depletion of *TCTP* mRNAs was induced through the addition of tetracycline (tet) and confirmed by western blot decorated with TCTP and EF1α antibodies (Figure 2A; Inset). After 24 hours of RNAi induction, no TCTP could be detected by western blot. Cell growth was monitored for five consecutive days (Figure 2A). Two days post RNAi induction the cells started to grow slower and the growth retardation became more severe on the following days. After five days of TCTP depletion the doubling time was prolonged from six to 24 hours, when compared to the non-induced cells. Phase contrast and DAPI images were used for cell morphology and cell cycle stage analysis (Figure 2B). Over the course of TCTP depletion, we observed an accumulation of cells in the 2K2N stage of the cell cycle. Quantification of kinetoplast-nucleus counts showed that after three days of RNAi induction the number of cells with 2K2N DNA content increases from 4% to 33% (n ≥ 100; Figure 2C). This corresponds to a decrease of cells in G1 of the cell cycle (1K1N) from 80% to 50% (Figure 2C). We did not observe a significant change in the number of 2K1N cells. Phase contrast images did not reveal any obvious change in the cell morphology upon TCTP depletion.

### TCTP is involved in heat-stress response in BSF parasites

Based on the reports in other model organisms we tested if TCTP in BSF cells is involved in stress response 20. For this we induced *TCTP* RNAi for 24 hours in bloodstream trypanosomes. At this time the levels of TCTP in BSF are below detection limit and no growth phenotype is observed (see Figure 2A). Following the depletion of TCTP the cells were exposed to heat stress for one hour, then the cells were washed with PBS and cultured in HMI-9 with 10% FCS without tetracycline. The recovery of cell growth was monitored in both cases by following cell growth for five consecutive days (Figure 2D). As controls we used cells that were heat stressed but not induced with tetracyclin as well as cells where RNAi was induced but that were not stressed. Additionally, we also used oxidative stress induced by NaAsO_2_ (Figure 2E) and heat stress in the PCF (Figure 2F). Average cumulative values from three independent experiments were plotted using GraphPrism with error bars indicating the standard deviation. We observed that in the control experiments the BSF cells grew at a wild type rate of approximately four cell divisions per day, while the BSF cells exposed to heat shock after *TCTP* RNAi showed a delayed recovery by two days. In the first 24 hours post heat shock the cells depleted of TCTP did not grow and a normal growth rate was first observed between 48 and 72 hours post heat shock (Figure 2D). The BSF cells exposed to oxidative stress following depletion of TCTP (Figure 2E) had only one cell division in the first 24 hours of the recovery but regained the normal growth rate (four divisions in 24 hours) two days after the stress was induced. In the PCF cells the heat stress delayed growth for 24 hours, however no significant difference between TCTP depleted cells and the wild type situation was observed. Based on these experiments we suggest that TCTP depletion in BSF cells has only a minor impact on recovery following oxidative stress while it is more pronounced under heat stress. In PCF cells recovery from heat stress does not seem to be influenced by the lack of TCTP.

### TCTP depletion does not alter mitochondrial or acidocalcisome morphology

**Figure 3:**
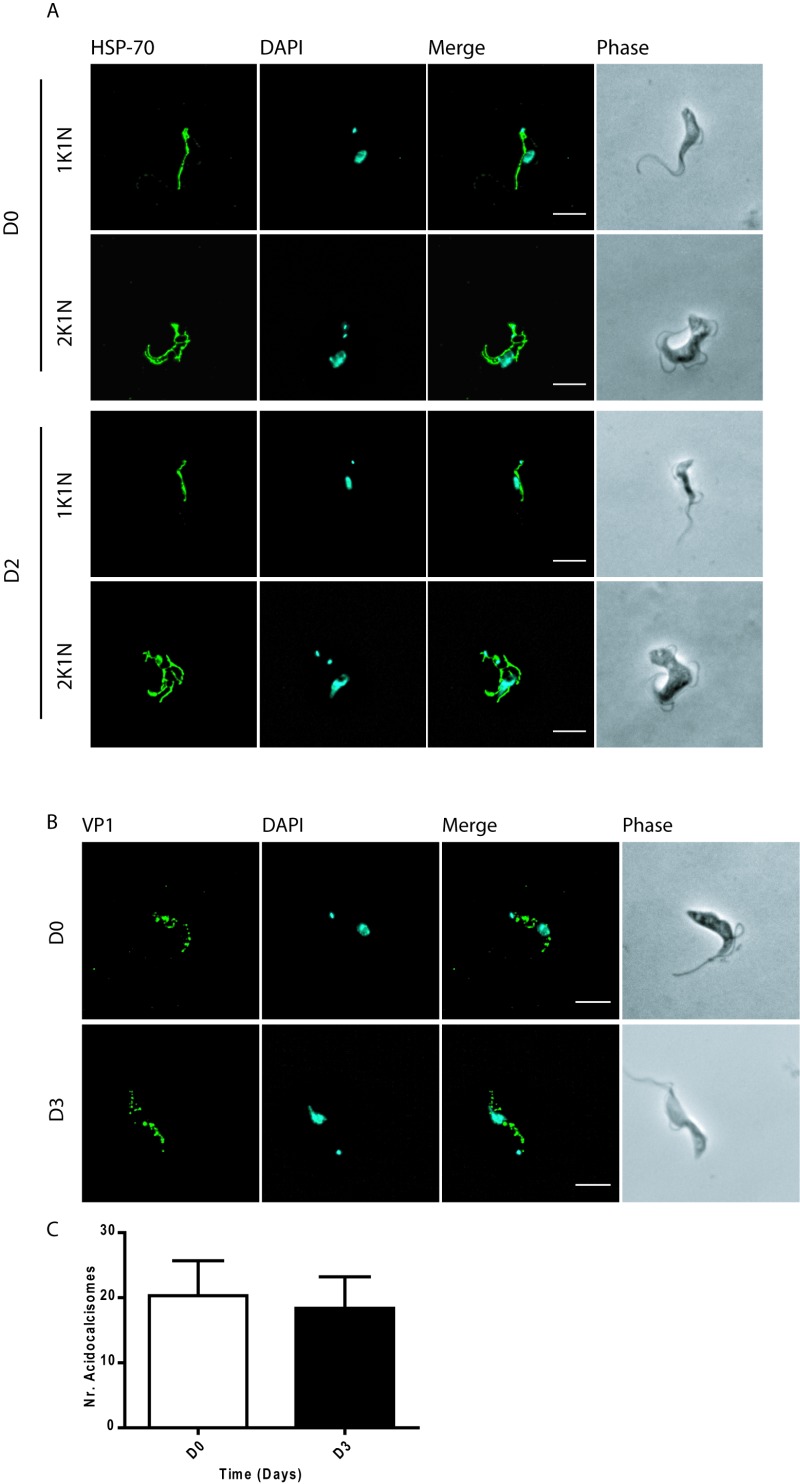
FIGURE 3: Effect of TCTP depletion on mitochondria and acidocalcisomes in BSF cells. (A) Immunofluorescence images of non-induced (D0) and induced at day two (D2) bloodstream cells stained for mitochondria (HSP-70, green). (B) Morphology of acidocalcisomes following downregulation of TCTP in BSF. Non-induced (D0) and induced at day three (D3) BSF trypanosomes were stained for acidocalcisome with the marker VP1 (green). Nuclear DNA and kDNA were detected with DAPI (cyan). Phase contrast images (grey) show cell morphology. Cell morphology is shown by phase contrast images (PH, grey). (C) Histograms showing the mean number of acidocalcisomes per cell in D0 and D3 cells (n = 13). Scale bars: 5 µm.

To verify if the depletion of TCTP in BSF trypanosomes would lead to mitochondrial structure abnormalities such as the ones observed in the PCF cells 13 we induced *TCTP* RNAi in BSF cells and visualized the mitochondria using the mitochondrial heat shock protein 70 (mtHSP-70) as a marker. Immunofluorescence images show no aberration in the mitochondrial structure upon two days of TCTP depletion (Figure 3A). Similarly, we asked if TCTP depletion in BSF cells would change the number and/or morphology of acidocalcisomes as we had previously observed in PCF cells. For this we visualized the acidocalcisomes by immunofluorescence microscopy using the vacuolar proton pyrophosphatase (VP1) antibody (Figure 3B, 21. The DNA and cell morphology were visualized by DAPI and phase contrast images. Following three days of TCTP depletion, we did not notice enlarged acidocalcisomes or a significant change in their number (Figure 3B, C).

### Identification of possible interactors and binding proteins of TCTP

In order to identify possible targets or interactors of TCTP in BSF trypanosomes we analysed the whole cell proteome before and after TCTP depletion using stable isotope labelling with amino acids in cell culture (SILAC) combined with mass spectrometry (Figure S1A-C, 22,23,24). Samples form non-induced and induced bloodstream trypanosomes were mixed in 1:1 ratio (cell number) and analyzed by liquid chromatography mass spectrometry. Overall, we could detect the changes of 2557 proteins. Proteome changes upon TCTP depletion for 24, 36 and 48 hours are presented by volcano plots (Figure S1A-C). Each plot represents the proteome comparison between non-induced cells and the respective time point of TCTP RNAi induction. In each plot the x-axes represent the proteins abundance change (log_2 _fold change) while the y-axes represent the significance of the observed fold change (log_10_ p-value). Marked in red is TCTP2. Over the course of TCTP RNAi induction, we observed a progressive depletion of TCTP2. We did not detect other proteins whose abundance changed progressively and significantly in all timepoints during the course of TCTP RNAi. Similarly, we did not identify any interaction partners of the PCF TCTP1 (Figure S2) when performing pull-down with the c-terminally HA-tagged version.

## DISCUSSION

We have previously reported that the *T. brucei *genome encodes two paralogues of the translationally controlled tumor protein and have named them TCTP1 and TCTP2 13. The two paralogues are differentially expressed during the parasite life cycle 13. TCTP2 is expressed in BSF cells and is repressed after the transition to the insect form parasites where the second paralogue *TCTP1* is almost exclusively expressed. Furthermore, we showed that the different 3' UTRs of the two paralogues are the basis of the mechanism controlling differential expression and characterized the morphological and organelle phenotypes in the insect form 13. Here, we study the function of TCTP in the BSF cells and compare them to the PCF providing a more comprehensive picture of TCTP in *T. brucei* (Table 1).

**Table 1:** Comparison of TCTP1 and TCTP2 in PCF and BSF parasites, respectively.

	**PCF**	**BSF**
**Expressed paralogue**	*TCTP1*	*TCTP2*
**Localization**	Cytoplasmic	Cytoplasmic
***TCTP*1/2 depletion by RNAi**		
**Cell growth**	Growth inhibition	Strong growth inhibition
**Cell cycle**	No change	Increase in 2K2N cells
**Cell morphology**	Tadpole morphology	No change
**Acidocalcisomes**	Enlarged in size and reduced in number	No change
**Mitochondria**	Accumulations in the network	No change
**+ heat stress**	No change	Decreased growth
**+ oxidative stress**	NA	No change
**Cell growth**	Growth inhibition	Strong growth inhibition

The localization of TCTP2 in the BSF is very similar to the insect form (Table 1). Based on biochemical and imaging data the protein is mostly distributed throughout the cytoplasm with a distinct depletion in the nucleus. Also, the localization does not change during the BSF cell cycle, similar to what we reported for the PCF trypanosomes 13. A cytoplasmic localization in trypanosomes is consistent with most reports from plant, yeast or mammalian systems 16,25. However, some reports have shown nuclear localization in human cells 26, as well as transient association with mitochondria and microtubules in yeast cells 16,25.

In order to test function of TCTP in BSF cells we used RNAi targeting the *TCTP* ORF. In previous experiments we specifically depleted the BSF TCTP2 by targeting the *TCTP2* 3’UTR, which led to a growth retardation, and while the overall TCTP levels remained below detection limit in western blotting, the exclusive depletion of *TCTP2* was accompanied by a temporary increase of the levels of *TCTP1* mRNA 13 (Table 1). Interestingly, RNAi experiments targeting the ORF of *TCTP*, thus also affecting the residual *TCTP1* expression, resulted in a much stronger growth inhibition of BSF cells. This supports the previously suggested hypothesis that the residual expression of TCTP1 in BSF cells might partially compensate for the specific loss of TCTP2.

In BSF cells we detect a specific increase in the 2K2N cells during *TCTP* ORF RNA, while specific depletion of TCTP2 leads to a slight increase in 1K1N cells (Figure S3) as was seen for the TCTP depletion in PCF cells. Thus, the specific depletion of the BSF *TCTP2* shows a different phenotype than depletion of both paralogues suggesting different functions of the two proteins. Alternatively, the ORF RNAi might be more efficient in depleting *TCTP*2 since it covers a larger region of the transcript, and this might lead to the different phenotypes.

The 2K2N cells have undergone mitochondrial and nuclear DNA replication as well as segregation but seem to be blocked or delayed in cytokinesis. Interestingly, this phenotype was not observed in the BSF specific *TCTP2 *RNAi knockdown (see Figure S3 and 13). Here a small increase in 1K1N cells similar to the PCF *TCTP* knockdown described previously was observed 13*. *While in BSF parasites cytokinesis is blocked or delayed, the cell division in PCF cells is unequal with one daughter inheriting a not fully developed posterior end leading to a "tadpole" like morphology (Table 1). A precytokinesis arrest similar to the one described above was previously observed by Sheader and coworkers upon silencing of VSG in BSF cells. Here the number of 2K2N cells reached up to 60% at 48 hours post induction 27. The authors speculated that this checkpoint which seems absent in the PCF cells is required *in vivo *to respond to potential changes in VSG levels during cell division 27.

For example, TCTP transiently interacts with microtubules and numerous cell cycle proteins including the polo-like kinese-1 28, the checkpoint protein Chfr 29, the mitotic regulator Cdc25C 8 and nucleolar proteins 30. These proteins interfere in different steps of the cell cycle resulting in a broad number of phenotypes when alternating the levels of TCTP. In this study we aimed to identify TCTP binding partners or effectors using SILAC proteomics after TCTP depletion. We only included timepoints before the growth phenotype appeared in order to avoid the detection of secondary effects. While we could clearly detect the loss of TCTP as a consequence of the* TCTP* RNAi we did not detect any other significant changes. Thus, TCTP might be involved in maintaining functionality or localization of its interacting partners rather than simply increasing the stability.

TCTP has been described as a calcium binding protein for the first time 20 years ago in trypanosomes 11 and later confirmed also in other organisms 31. We recently demonstrated that TCTP depletion in PCF parasites causes alterations in the morphology of the major calcium storage organelles, acidocalcisomes and mitochondria 13. In BSF cells both organelles seem not affected by the loss of TCTP, while the growth defect remained. Thus, potentially the effect on the calcium storage organelles in PCF are due to secondary effects.

One of the best studied and conserved functions of TCTP is the ability to protect cells against a vast number of cellular stresses. During heat shock, an elevated level of TCTP has been observed in worm parasites such as *Schistosoma* and *Trichinella* 6,7. This has been hypothesised to act as a stress defence mechanism to protect the parasite during the transition from the cold-blooded vectors (snails) to the worm blooded hosts (vertebrates). On the other hand, silencing of the gene using RNAi led to decreased tolerance to cold and high temperatures in the *Brassica oleracea* cabbage 5. Here we tested whether the presence of TCTP helps in cell recovery following heat or oxidative stresses. For this we depleted TCTP in BSF cells and subjected them to the heat or oxidative stress, followed by monitoring the cell growth recovery. We found that the cells in which TCTP was depleted prior to the heat stress survive the stress but recover with a delay of two days compared to the controls. Interestingly this result was observed only in bloodstream cells and not in PCF indicating that stress response might be specific for that life cycle stage. Following the mild oxidative stress, both non-induced and induced cells recovered at similar rates. Overall, these data suggest that endogenous levels of TCTP are required for the bloodstream forms recovery following heat stress. TCTP might be chaperoning other proteins and/or mRNAs which help cellular recovery, a model already suggested in the worm parasites 6.

## MATERIALS AND METHODS

### Trypanosome cell lines and culturing

For RNAi and gene tagging experiments we used transgenic *T. brucei* bloodstream (New York single marker, NYsm) cell lines co-expressing T7 RNA polymerase and a tet repressor 32. The BSF cells were cultured at 37°C and 5% CO_2_ in HMI-9 medium supplemented with 10% FCS 33 in the presence of 2.5 µg/ml geneticin (G418).

### Plasmid constructs and transfection

For inducible RNAi against *TCTP* mRNAs we used a pLEW100 based stem-loop plasmid 32,34 where an insert of 512 bp targeting the full ORF sequence of the TCTP2 gene was integrated. The constructs were linearized with NotI and 10 µg were transfected in NYsm BSF by electroporation. The positive clones were selected with blasticidin (2.5 g/ml in BSF). Induction of RNAi was done by addition of 1 µg/ml tet. For the C-terminal tagging one allele of TCTP2 in BSF was *in situ* tagged with a triple Hemagglutinin (HA) epitope 35. For inducible N-terminal c-Myc tagging, the full ORF plus the first 21 nt from the 3’UTR of TCTP2 were amplified by PCR and cloned in pJM-2 vector (a gift from A. Schneider; 34. Upon transfection (as described above) the clones were selected with puromycine. Expression was induced by addition of 1 μg/ml tet.

### Growth recovery assay

Downregulation of TCTP in BSF and PCF was induced for 24 hours. The cells were then washed twice in PBS and subjected to either heat or oxidative stress. For the heat shock, the cells were pelleted by centrifugation (2500 rpm/8 min), re-suspended and incubated in pre-warmed media (one hour at 42°C HMI-9 for BSF, 45 minutes at 41°C SDM-79 for PCF). Oxidative stress was induced by incubating BSF for 3 hours in HMI-9 media supplemented with 50 µM sodium arsenate, NaAsO_2 _36. Immediately after each stress the cells were washed in PBS and re-cultured in their normal growth conditions. Their recovery was monitored by counting cell growth in the next four or five consecutive days. Average cumulative values from three independent experiments were plotted using GraphPrism with error bars indicating the standard deviation.

### Western blot

For western blotting trypanosome pellets were washed in phosphate buffered saline (PBS, pH = 7.2), re-suspended in standard Laemmli buffer (LB) (10^6^ in 15 µl), boiled for 5 min at 95°C and cooled on ice for 5 minutes. For the digitonin fractionation the cells were washed once in PBS, then the pellets were re-suspended in SoTE buffer (0.6 M sorbitol, 2 mM EDTA, 20 mM Tris-HCl, pH 7.5) containing 0.025% digitonin and incubated on ice for 5 min. The cell fractions were then separated by differential centrifugation at 8000 rcf for 5 min at 4°C. The fractions were lysed in LB, boiled for 5 min at 95°C, cooled on ice for 5 minutes and loaded on 10% or 12% SDS-polyacrylamide gels (10^6^ - 10^7^ cells per lane) before subjected to western blotting. The proteins were transferred onto PVDF Immobilon-P membranes (Millipore) using BioRad wet blotting system, blocked for 1 hour at room temperature in 10% skimmed milk or BSA solution in PBST (PBS + 0.1% TWEEN-20) and decorated with the primary antibodies. In this study we used rat-polyclonal anti-TCTP (1:50, Eurogentech), rabbit anti-ATOM (1:10000, 37) and mouse anti-EF1alpha (1:10000, SantaCruz). After washing the primary antibody by incubating the membranes 3 times, 10 minutes each time in PBST, the membranes were incubated for 1 hour at room temperature with the secondary antibody. Secondary antibodies were: rabbit anti-rat HRP-conjugate (1:10000, Dako), swine anti-rabbit HRP-conjugate (1:10000, Dako) and rabbit anti-mouse HRP-conjugate (1:10000, Dako). For the chemiluminescent detection, the SuperSignal system (Pierce) was used and images were acquired with Amersham Imager 600.

### Immunofluorescence and microscopy

For immunofluorescence, BSF were harvested by slow centrifugation (2000 rpm/5 min), washed once in PBS and then fixed on slides for 4 min with 4% PFA in PBS. The cells were permeabilized for 5 min with 0.2% TritonX-100 in PBS and blocked for 30 min with 4% BSA in PBS. The cells were incubated for 1 hour in a wet chamber with primary antibody diluted in 4% BSA in PBS, washed three times in PBST and incubated again in dark wet chambers with secondary antibodies diluted in 4% BSA in PBS. The cells were mounted with ProLong® Gold Antifade Mountant with or without DAPI (Invitrogen). Images were acquired with Leica DM 5500 fluorescent light microscope and/or Leica SP8 Confocal Microscope System with STED and deconvoluted by Leica LAS AF and Huygens software, respectively. The primary antibodies used in this study were: rabbit anti-myc (1:1000, Sigma), rabbit anti-HA (1:1000, Sigma), mouse anti-mtHSP70 (1:2000, 38) and rabbit anti-VP1 (1:2000, 21). Secondary antibodies were goat anti-rabbit IgG, goat anti-mouse IgG conjugated with fluorophores Alexa Fluor® 488, Alexa Fluor® 594 (1:1000, Invitrogen).

## SUPPLEMENTAL MATERIAL

Click here for supplemental data file.

All supplemental data for this article are also available online at http://microbialcell.com/researcharticles/the-translationally-controlled-tumor-protein-tctp-is-involved-in-cell-cycle-progression-and-heat-stress-response-in-the-bloodstream-form-of-trypanosoma-brucei/.
