# Supplementary material for: The translationally controlled tumor protein TCTP is involved in cell cycle progression and heat stress response in the bloodstream form of Trypanosoma brucei
Source: Microb Cell. 2018 Aug 24;5(10):460–8. doi: 10.15698/mic2018.10.652 (PMC6206406; doi:10.15698/mic2018.10.652)
Supplement: Supplementary file 1 [file mic-05-460-s01.pdf]

Supplementary information

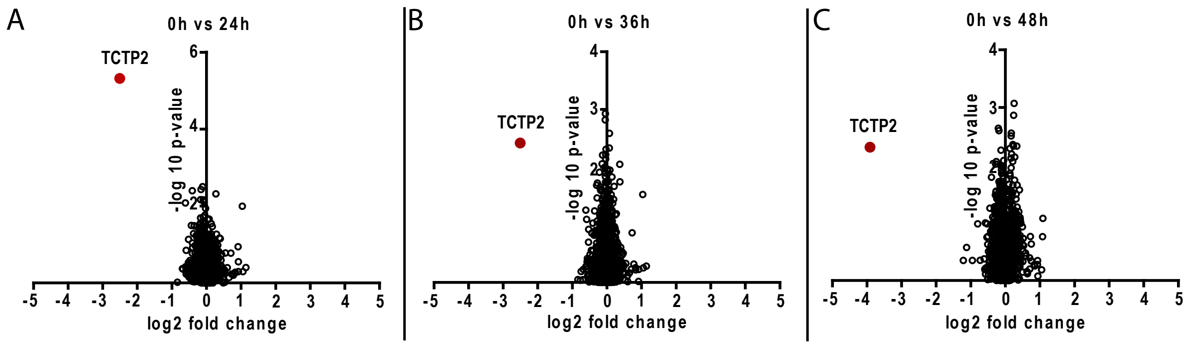

Figure S1 Volcano plots showing log_2 fold changes of protein abundances following TCTP RNAi in bloodstream trypanosomes. The proteomes of non-induced cells were compared with the ones where downregulation of TCTP was induced for (A) 24 hours, (B) 36 hours (C) or 48 hours. For each time point measurements were done in triplicates. p-values were calculated using two tailed student's t-test.

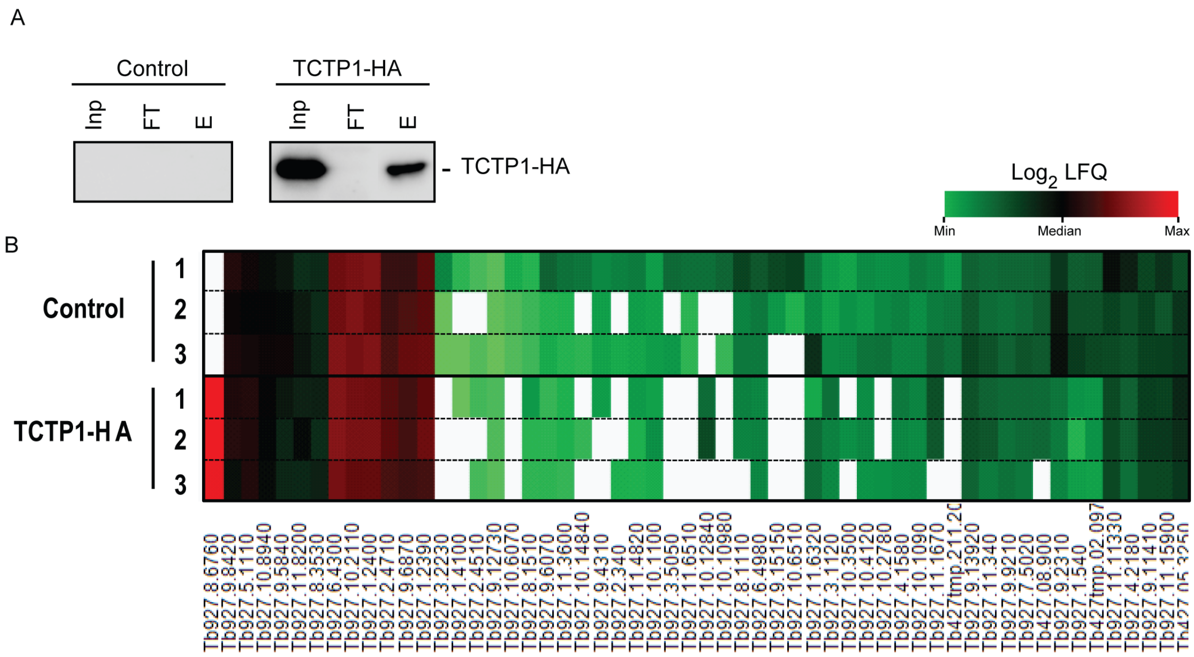

Figure S2 Co-immunoprecipitation of TCTP1-HA in procyclic trypanosomes. (A) Lysate of procyclic trypanosomes in which TCTP1 was c-terminally tagged with HA was incubated with anti-HA conjugated magnetic beads. Input (inp), flowthrought (FT) and eluate (E) were analyzed by Western blot decorated with anti-HA antibody. Equivalent cell numbers were loaded in each lane. Non-tagged procyclic 29-13 were used as control for specificity. (B) Table showing mass spectrometry analysis results of TCTP bound anti-HA conjugated beads. Color coded are displayed the log2 label-free quantifiers (LFQ) calculated by MaxQuant on protein intensity. Color code is relative to the highest and lowest log2 transformed LFQ value with brightest red being the maximum, brightest green the minimum, black the median. In white are missing values.

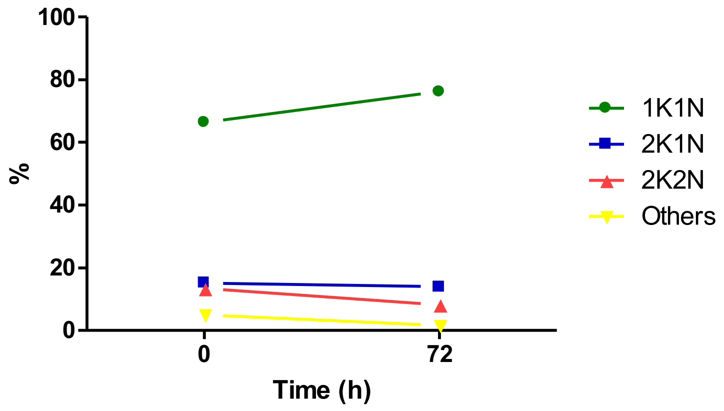

Figure S3 DNA content analysis following TCTP ORF RNAi in BSF cells (n ≥ 100). Two timepoints are shown 0 and 72h post RNAi induction. 1K1N, cells with one kinetoplast and one nucleus.
